# SIRT2 inhibitor SirReal2 enhances anti‐tumor effects of PI3K/mTOR inhibitor VS‐5584 on acute myeloid leukemia cells

**DOI:** 10.1002/cam4.6480

**Published:** 2023-09-01

**Authors:** Yiming Luo, Haijun Zhao, Jingtao Zhu, Liyi Zhang, Jie Zha, Li Zhang, Yi Ding, Xinyi Jian, Junjie Xia, Bing Xu, Zhongquan Qi

**Affiliations:** ^1^ Department of Hematology The First Affiliated Hospital of Xiamen University and Institute of Hematology, School of Medicine, Xiamen University Xiamen Fujian China; ^2^ Key Laboratory of Xiamen for Diagnosis and Treatment of Hematological Malignancy Xiamen Fujian China; ^3^ The School of Clinical Medicine Fujian Medical University Fuzhou Fujian China; ^4^ Department of Gastrointestinal Oncology Surgery, Cancer Center The First Affiliated Hospital of Xiamen University Xiamen Fujian China; ^5^ The Third Clinical Medical College Fujian Medical University Fuzhou Fujian China; ^6^ Department of Breast Surgery, Key Laboratory of Breast Cancer in Shanghai Fudan University Shanghai Cancer Center Shanghai China; ^7^ Department of Oncology Fudan University Shanghai Medical College Shanghai China; ^8^ Department of Pathology, The First Affiliated Hospital, School of Medicine Xiamen University Xiamen China; ^9^ Graduate College of Fujian Medical University Fuzhou Fujian China; ^10^ Organ Transplantation Institute of Xiamen University Xiamen Fujian China; ^11^ Fujian Provincial Key Laboratory of Organ and Tissue Regeneration Xiamen Fujian China; ^12^ Xiamen Key Laboratory of Regeneration Medicine School of Medicine, Xiamen University Xiamen China; ^13^ Medical College of Guangxi University Nanning Guangxi China

**Keywords:** acute myeloid leukemia, anti‐tumor effect, PI3K/mTOR, SIRT2

## Abstract

**Background:**

Acute myeloid leukemia (AML) is a highly aggressive form of cancer that is frequently diagnosed in adults and small molecule inhibitors have gained significant attention as a potential treatment option for AML.

**Methods:**

The up‐regulated genes in AML were identified through bioinformatics analysis. Potential candidate agents were selected through pharmacogenomics analysis. Proteomic experiments were conducted to determine the molecular mechanism after inhibitor treatment. To evaluate drug synergy, both cellular functional experiments and an AML mouse model were used.

**Results:**

Through bioinformatics analysis, we conducted a screening for genes that are highly expressed in AML, which led to the identification of nine small‐molecule inhibitors. Among these inhibitors, the PI3K/mTOR inhibitor VS‐5584 demonstrated significant effectiveness in inhibiting AML cell proliferation at low concentrations. Further testing revealed that VS‐5584 induced apoptosis and cycle arrest of AML cells in a dose‐ and time‐dependent manner. Proteomics analysis showed significant changes in protein expression profiles of AML cells after VS‐5584 treatment, with 287 proteins being down‐regulated and 71 proteins being up‐regulated. The proteins that exhibited differential expression were primarily involved in regulating the cell cycle and apoptosis, as determined by GO analysis. Additionally, KEGG analysis indicated that the administration of VS‐5584 predominantly affected the P53 and SIRT2 signaling pathways. The use of SIRT2 inhibitor SirReal2 alongside VS‐5584 caused a significant reduction in the half‐maximal inhibitory concentration (IC_50_) of VS‐5584 on AML cells. In vivo, experiments suggested that VS‐5584 combined with SirReal2 suppressed tumor growth in the subcutaneous model and extended the survival rate of mice injected with tumor cells via tail vein.

**Conclusions:**

Taken together, the PI3K/mTOR inhibitor VS‐5584 was effective in suppressing AML cell proliferation. PI3K/mTOR inhibitor combined with SIRT2 inhibitor exhibited a synergistic inhibitory effect on AML cells. Our findings offer promising therapeutic strategies and drug candidates for the treatment of AML.

## 1 INTRODUCTION

Acute myeloid leukemia (AML) is a common hematological malignancy,^1^, ^2^ with a five‐year survival rate of only 24%.^3^, ^4^, ^5^ Despite significant advancements in therapeutic approaches for AML, including successful clinical cures for certain types such as M3 AML,^6^, ^7^ many patients still have a poor prognosis.^8^, ^9^ This is reflected in the tens of thousands of AML‐related deaths that occur annually in China,^10^ which places a considerable burden on both families and society.

Acute Myeloid Leukemia (AML) is a highly heterogeneous disease, with varying development patterns among individuals.^11^ Despite extensive research on its pathogenesis, current therapeutic strategies remain inadequate. Therefore, there is an urgent need to develop new and effective drugs to treat AML. To address this, a search of publicly available databases to identify genes that exhibit significant upregulation in AML. Subsequently, we screened small molecule inhibitors corresponding to these genes using pharmacogenomics. Through cellular function assays, we determined that the PI3K/mTOR inhibitor VS‐5584 exhibited the highest anti‐tumor effect. The phosphatidylinositol 3‐kinase/protein kinase B/mammalian target of rapamycin (PI3K/AKT/mTOR) signaling pathway plays a crucial role in various cellular processes such as transcription, translation, cell cycle, cell differentiation, and apoptosis.^12^ The PI3K/AKT/mTOR signaling pathway is frequently dysregulated in different types of cancer, including hematologic malignancies.^13^ This continuous activation of the pathway causes an imbalance in apoptosis regulation and excessive cell proliferation, ultimately leading to the growth of tumor cells. Several studies have reported that the PI3K/AKT/mTOR pathway is excessively activated in more than 50% of cases of AML, up to 88% of cases of ALL, and also in cases of chronic myeloid leukemia (CML) and chronic lymphocytic leukemia (CLL).^14^, ^15^

While PI3K/AKT/mTOR inhibitors have shown promise in treating solid tumors,^16^ their use in hematologic malignancies is not yet common. In vitro, experiments have demonstrated their ability to inhibit leukemia cells, but clinical trials evaluating their efficacy in vivo have had limited success. It is crucial to explore the efficacy of PI3K/AKT/mTOR inhibitors in leukemia animal models through in vivo experiments. The single‐agent activity of PI3K/AKT/mTOR inhibitors is relatively limited.^17^ Therefore, future research should focus on the utilization of effective dual inhibitors and exploring other combinations with leukemia‐related pathway inhibitors. The treatment of leukemia can be significantly improved through the scientific and rational combination of drugs.^18^, ^19^, ^20^, ^21^ This approach has been shown to offer high efficacy while also minimizing tolerance issues during the treatment process, making it a promising area of research for further exploration.

In this study, we utilized bioinformatics to analyze the differentially expressed genes that were significantly upregulated in AML and had a negative correlation with patient survival. Through pharmacogenomics analysis, inhibitors of the up‐regulated genes were screened. The results showed that the PI3K/mTOR inhibitor VS‐5584 effectively suppressed AML cell proliferation. The impact of VS‐5584 on the SIRT2 signaling pathway was found to be significant. Moreover, when combined with a SIRT2 inhibitor, VS‐5584 demonstrated a synergistic inhibitory effect on AML cells. These findings suggest that both VS‐5584 and SIRT2 inhibitors have the potential to be effective drug candidates for the treatment of AML.

## 2 MATERIALS AND METHODS

### 2.1 Bioinformatics analysis

The adult acute myeloid leukemia (LAML) dataset was derived from The Cancer Genome Atlas data portal (https://tcga‐data.nci.nih.gov/). To validate our findings, we also utilized the BEAT AML database (http://vizome.org/aml/). RNA‐Seq datasets from GEPIA.22 were used to generate survival curves for AML patients.^22^ The study identified differentially expressed genes that were significantly upregulated in AML and selected them for further analysis. Gene ontology analysis was conducted using GeneCodis, while pathway analysis was determined using Kyoto Encyclopedia of Genes and Genomes (KEGG) pathway annotations. Additionally, a protein–protein interaction network was performed using STRING, which included both direct (physical) and indirect (functional) interactions.

### 2.2 Drug‐gene interaction

To analyze drug‐gene interaction, we utilized the DGIdb database (http://dgidb.genome.wustl.edu/). This database is a comprehensive resource that provides information on drug‐gene interactions and the druggable genome. The data is derived from over 30 reliable sources, such as DrugBank, PharmGKB, ChEMBL, NCBI Entrez, Ensembl, and PubChem.

### 2.3 Cell Culture

Human THP1, KG1a, and Kasumi‐1 leukemia cell lines were obtained from the Chinese Academy of Sciences. AML cells were cultured in DMEM/high glucose medium containing 10% FBS and 1% penicillin–streptomycin solution and kept in a humidified incubator at 37°C with 5% carbon dioxide.

### 2.4 CCK8 assay

AML cells (1000 cells per well) were treated with inhibitors for the desired duration. Afterward, the CCK8 solution was added and incubated for 2 h. The absorbance at 450 nm was measured using a Microplate Reader (Bio‐Rad Laboratories, Inc.).

### 2.5 Cell apoptosis assay

The Annexin V‐FITC Apoptosis Detection Kit was obtained from Beyotime Biotechnology (#C1062L) in China and used to stain AML cells according to the manufacturer's instructions. Cell apoptosis was evaluated using flow cytometry and the resulting data were analyzed using Flowjo software (FlowJo LLC).

### 2.6 Cell cycle assay

AML cells treated with inhibitors were fixed with pre‐chilled 70% ethanol overnight. After washing with PBS, the cells were stained with propidium iodide solution at 37°C for 30 min. The cell cycle distributions were examined using a Flow Cytometer, and the data were processed with FlowJo.

### 2.7 Western blot analysis

Proteins were extracted from AML cells or tissues using the cOmplete Lysis Kit (Roche) and their concentrations were estimated using Bradford's method. For immunoblotting, an equal amount of protein samples was fractionated by SDS‐PAGE electrophoresis and transferred onto PVDF membranes. The membranes were blocked in TBST with 5% skim milk for 2 h. Primary antibody was added and incubated overnight at 4°C, followed by incubation with secondary antibody for 1.5 h at room temperature. The immunoblot band intensity was quantified using ImageJ software, with GAPDH detection used as a loading control.

### 2.8 Proteomics analysis

AML cells were collected subsequent to digestion and centrifugation. The resultant cell pellets were resuspended in 0.5 mL of SDT‐lysis buffer, comprising 100 mM DTT, 4% SDS, and 100 mM Tris–HCl. The protein samples were mixed and subjected to incubation at 100°C in a water bath for 10 min, followed by cooling to room temperature. The supernatant was subsequently collected through centrifugation, and the protein concentration was determined using the BCA method. Based on the protein concentration, 200 μg of protein was pipetted and placed on ice. A 5 μL solution of DTT was then added to the tube, which was subsequently incubated at 50°C for 10 min. Following this, 5 μL of IAA solution was added and thoroughly mixed. The tubes were then wrapped in aluminum foil to protect them from light and left at room temperature for 30 min. Subsequently, 5 μg of pancreatin powder was meticulously added and mixed with the solution. The resulting mixture was subjected to overnight incubation in a water bath maintained at 37°C. The supernatant was obtained through centrifugation, and 5 μg of Strata‐X was subsequently introduced to the supernatant to facilitate protein desalting.

Next, the supernatant underwent analysis via liquid chromatography–tandem mass spectrometry (LC–MS). To achieve this, the samples were dissolved in a 0.1% aqueous formic acid solution and introduced into a C18 column (100 μm*20 mm, 5 μm, Germany) for reversed‐phase high‐performance liquid chromatography (RP‐HPLC) at a flow rate of 300 nL/min. The mobile phase A comprised a 0.1% formic acid aqueous solution, while the isocratic solution containing 95% acetonitrile constituted mobile phase B. Peptide elution was accomplished using mobile phase B over a period of 20 min.

The acquisition of MS data was conducted through the utilization of a data‐dependent Top20 method, which involved the dynamic selection of precursor ions with the highest abundance from the survey scan (300–1800 m/z) for fragmentation via higher‐energy collision‐induced dissociation (HCD). Internal calibration was achieved through the implementation of the lock mass option (m/z 445.120025). Additional instrument parameters were established as follows: MS/MS resolution was set at 17500 at 200 m/z; the maximum injection time was limited to 50 ms; the normalized collision energy was set at 27%; the isolation window was established at 1.6 Th; and dynamic exclusion was set at 60 s.

The acquired raw data underwent processing through the utilization of MaxQuant software v1.6.0.16. The precursor ion tolerance was established at 20 parts per million (ppm), while the fragment ion tolerance was set at 4.5 ppm. Cysteine aminomethylation was designated as a fixed peptide modification, and protein amino‐terminal acetylation and methionine oxidation were set as variable modifications. The false‐discovery rate (FDR) was maintained at <1%. Protein abundance was determined through label‐free quantification (LFQ) values, and the MaxLFQ algorithm integrated into the MaxQuant software was utilized for protein quantification. Proteins were deemed to be significantly differentially expressed if they met two criteria: a p‐value for differential expression less than 0.05 and a fold‐change >1.5.

### 2.9 Tumor xenograft model of nude mouse

Male BALB/c nude mice (nu/nu) aged 6 weeks were housed in a specific pathogen‐free (SPF) room and provided with ad libitum access to food and water. Prior to their inclusion in the study, all mice were allowed to acclimate to the environment for a minimum of 1 week. THP1 cells in logarithmic growth phase were harvested and dispensed into 1.5 mL microcentrifuge tubes. The skin on both sides of the chest of male nude mice was sterilized with betadine solution after they were anesthetized. A 100 μL cell suspension containing 2 × 10^6^ cells was subcutaneously injected into the mice. Upon reaching a tumor volume of approximately 100 mm^3^, the subcutaneous tumor model was subjected to drug administration of 4 mg/kg of VS‐5584 and/or 4 mg/kg of SirReal2 via intraperitoneal injection. The drugs were administered every 3 days until the mice were sacrificed. Additionally, further experiments were conducted to investigate the impact of VS‐5584 and SirReal2 on tumor growth using a tail vein injection model. Intravenous injection of THP1 cells (1 × 10^6^ per mouse) was performed, followed by intraperitoneal injection of 4 mg/kg of VS‐5584 and/or 4 mg/kg of SirReal2 every 3 days until the mice were euthanized. All animal experiments were approved by the Animal Ethics Committee of The First Affiliated Hospital of Xiamen University.

### 2.10 Statistical analysis

The data were analyzed using SPSS 22.0 statistical software. The Shapiro–Wilk test was employed to assess the normality of the data distribution. In instances where the experimental data exhibited normal distribution characteristics, the Student's *t*‐test was utilized to compare two groups, while one‐way or two‐way ANOVA was employed to compare more than two groups. Data were expressed as mean ± standard deviation. Conversely, when the data did not conform to normal distribution characteristics, the Wilcoxon signed‐rank test was applied. The survival of mice was determined by Kaplan–Meier analysis.

## 3 RESULTS

### 3.1 Up‐regulated genes in AML and potential drugs discovered based on drug–gene interaction

**FIGURE 1:**
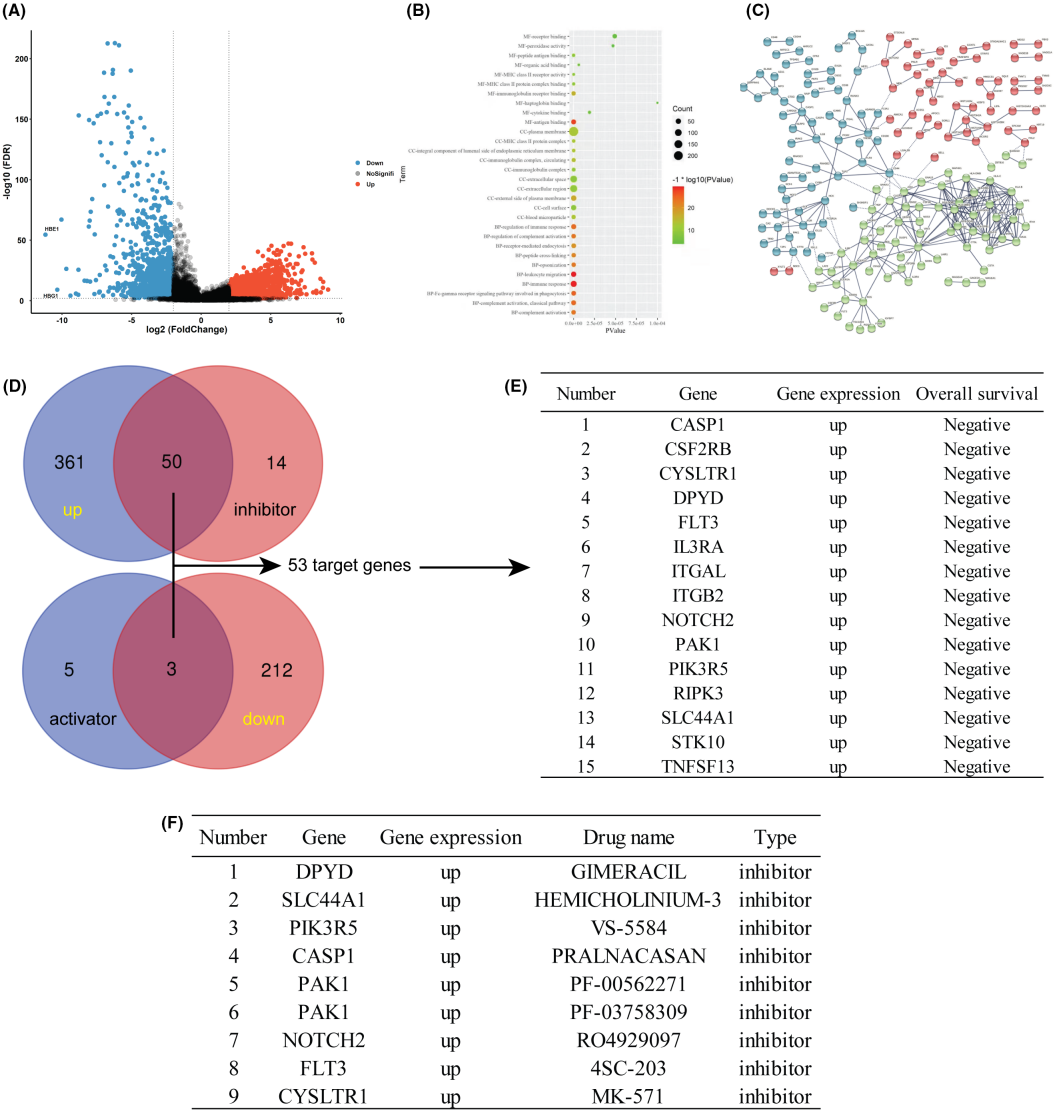
Genes significantly upregulated in AML and the pharmacogenomic analysis. (A) Volcano plot illustrating the differentially expressed genes in AML. (B) GO enrichment analysis of differentially expressed genes in AML. (C) Protein–protein interaction analysis of differentially expressed genes in AML. (D) Schematic diagram of the pharmacogenomic analysis of differentially expressed genes. (E) Genes associated with AML survival. (F) Small‐molecule inhibitors of target genes. AML, acute myeloid leukemia cells; GO, gene ontology.

Using bioinformatics, we analyzed the gene expression differences between normal donor bone marrow and bone marrow samples from patients with AML. Our findings indicated that out of 2500 genes, 1200 genes were up‐regulated and 1300 genes were down‐regulated in the AML samples (Figure 1A). GO analysis revealed that the differentially expressed genes were mainly involved in caspase binding, GTPase activity, and protein tyrosine phosphatase activity (Figure 1B). PPI analysis demonstrated significant clustering of the differential genes (Figure 1C). Furthermore, our search of the drug‐gene interaction database identified 50 genes with inhibitors that were up‐regulated and 3 genes with activators that were down‐regulated (Figure 1D). The survival analysis on 53 genes showed that 15 genes were up‐regulated in bone marrow samples of AML patients and were found to have a negative correlation with patient survival (Figure 1E; Figure S1). Consequently, these genes were categorized as oncogenes in AML. Subsequent examination revealed that 9 of the 15 genes possess the potential to be developed into candidate drugs for future use (Figure 1F). To further validate the transcriptional levels of these eight differential genes in AML, we utilized the BEAT AML database. The results were consistent with our findings, indicating that *CASP1, CYSLTR1, DPYD, FLT3, NOTCH2, PAK1, PIK3R5*, and *SLC44A1* were significantly upregulated in AML in comparison to the control (Figure S2).

### 3.2 Multiple small‐molecule drugs impeded AML cell proliferation

**FIGURE 2:**
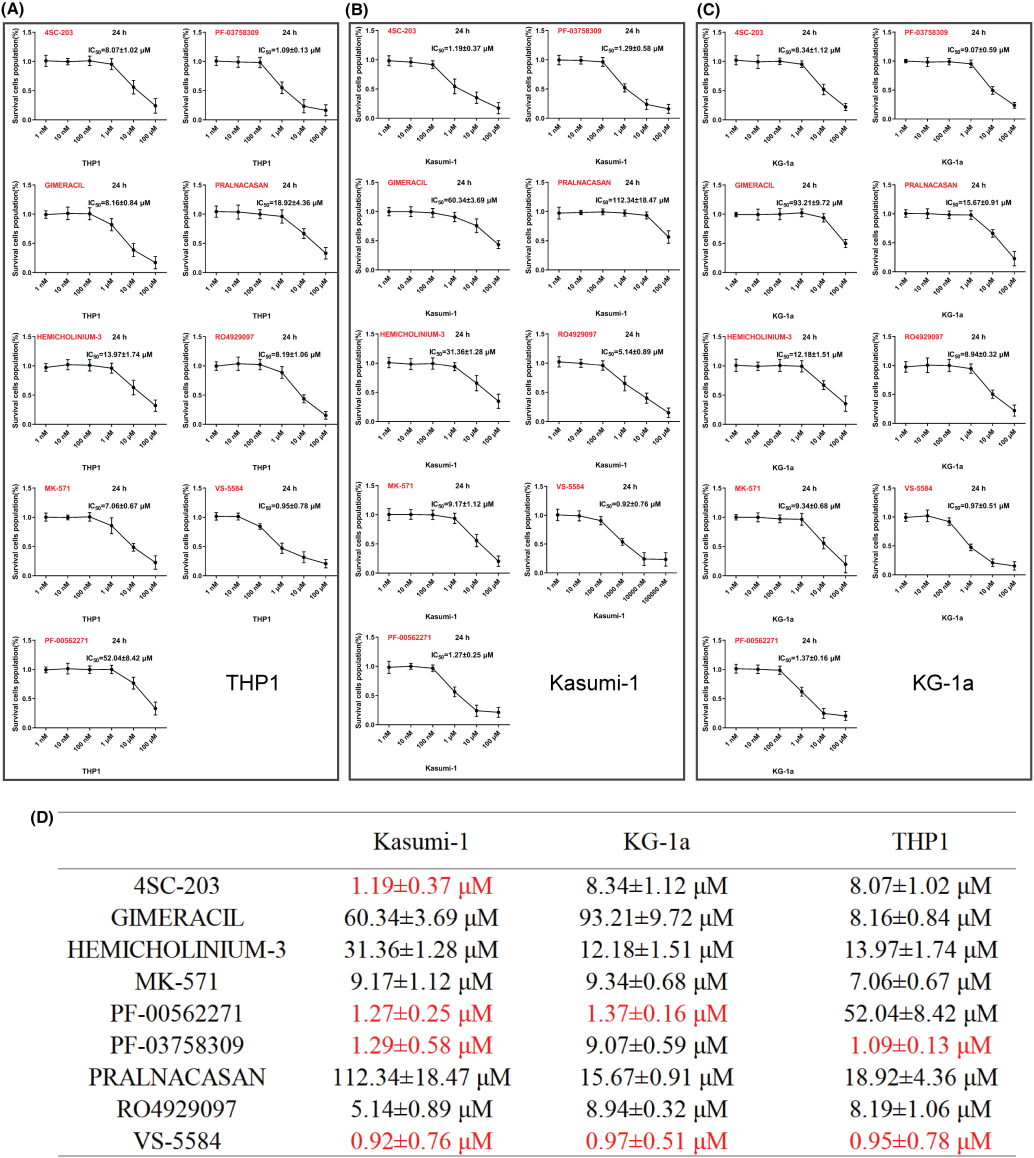
Multiple small‐molecule drugs inhibited AML cell proliferation. (A–C) Effects of small‐molecule inhibitors on the proliferative capacities of (A) THP1, (B) Kasumi‐1, and (C) KG‐1a cells. (D) The IC_50_ values of small‐molecule inhibitors on three types of AML cells. AML, acute myeloid leukemia cells.

In this study, three types of AML cell lines Kasumi‐1, KG‐1a, and THP1 were used to investigate the biological activities of nine inhibitors. The findings of this study confirmed that the aforementioned small molecule inhibitors exhibited significant killing effects on AML cells, with 4SC‐203, PF‐00562271, PF‐03758309, and VS‐5584 demonstrating strong inhibitory effects on AML cell proliferation (Figure 2A–C). Specifically, the IC_50_ of 4SC‐203 was determined to be 1.75 ± 0.37 μM, while the IC_50_ of PF‐00562271 for Kasumi‐1 and KG‐1a were 1.27 ± 0.25 nM and 1.37 ± 0.16 μM, respectively. The IC50 values of PF‐03758309 on Kasumi‐1 and THP1 cells were found to be 1.29 ± 0.58 μM and 1.09 ± 0.13 μM, respectively. Similarly, the IC_50_ values of VS‐5584 against Kasumi‐1, KG‐1a, and THP1 cells were determined to be 0.92 ± 0.76 μM, 0.97 ± 0.51 μM, and 0.95 ± 0.78 μM, respectively (Figure 2D).

### 3.3 VS‐5584 treatment contributed to G2/M cell cycle arrest in AML cells

In light of the high sensitivity of AML cell lines to VS‐5584, we proceeded to further investigate the biological activity of this compound in AML cells. Considering that VS‐5584 functions as a small molecule inhibitor that targets PI3K/mTOR, we tested the content of crucial molecules within the PI3K/AKT downstream pathway by Western blotting assay. Our findings indicated that treatment with 0.5 μM VS‐5584 resulted in a significant reduction of p‐mTOR levels in AML cells, as well as a significant decrease in the phosphorylation levels of its direct downstream targets, S6 Kinase 1 (S6K1) and eukaryotic translation initiation factor 4E‐binding protein (4EBP). S6K1 was observed to phosphorylate and facilitate the degradation of programmed cell death 4 (PDCD4) (Figure S3). These results confirmed that VS‐5584 treatment effectively suppressed the PI3K/AKT pathway in AML cells, resulting in alterations in the expression of downstream signaling molecules.

**FIGURE 3:**
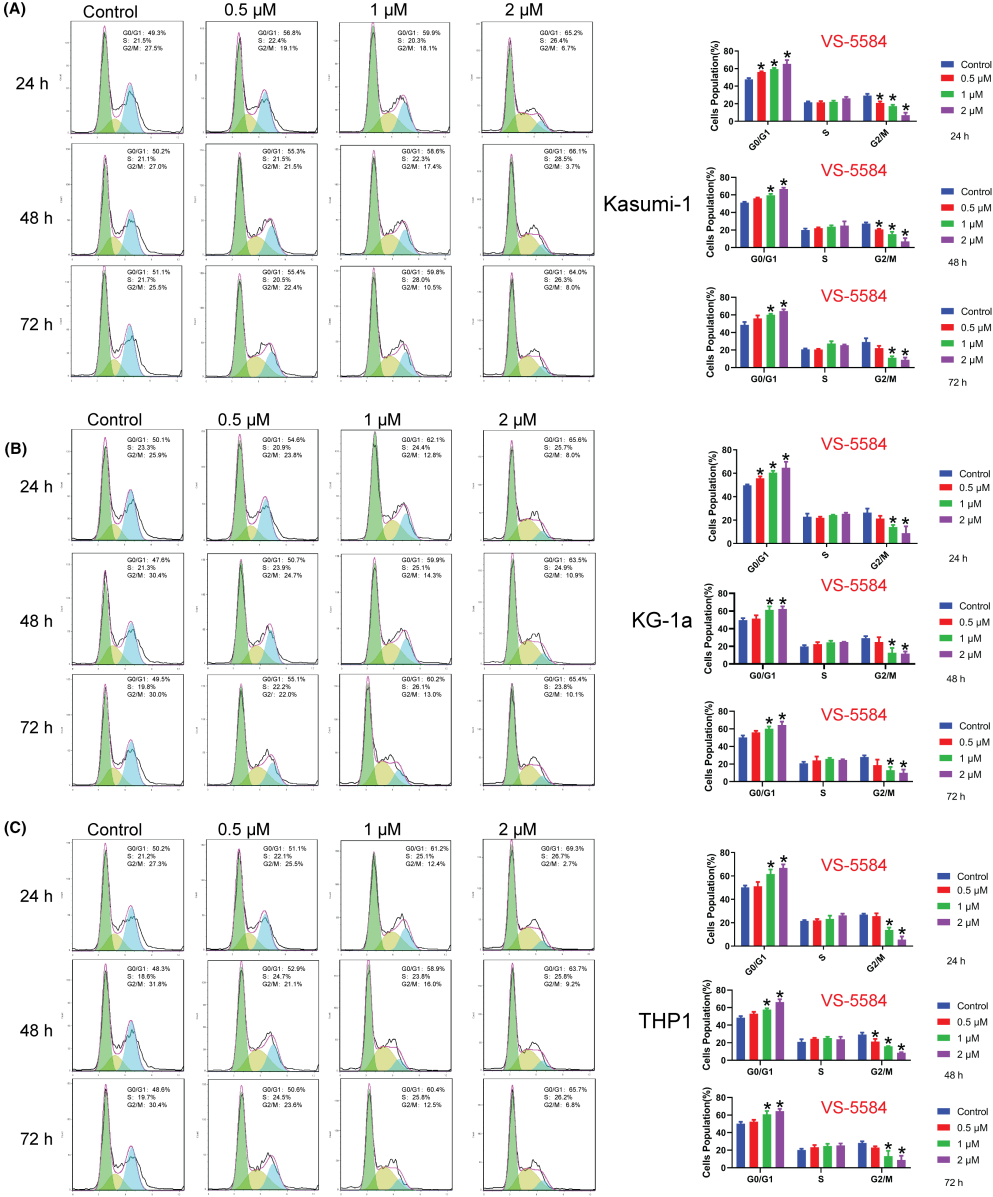
VS‐5584 treatment induced AML cell cycle arrest. (A–C) Various concentrations of VS‐5584 led to G2/M cell cycle arrest in (A) Kasumi‐1, (B) KG‐1a, and (C) THP1 cells. The treatment duration was 24, 48, or 72 h for the AML cells. *p<0.05. AML, acute myeloid leukemia cells.

We selected concentrations in the vicinity of the IC_50_ of VS‐5584 for subsequent experiments, with time points set at 24 h, 48 h, and 72 h. Our findings indicated that VS‐5584 induced cell cycle arrest at the G2/M phase. However, we observed no significant effect on cell‐cycle distribution in Kasumi‐1, KG‐1a, and THP1 cell lines at a concentration of 0.5 μM. Notably, higher concentrations of VS‐5584 (1 μM and 2 μM) were found to arrest cell cycle progression (Figure 3A–C).

### 3.4 VS‐5584 treatment induced apoptosis in AML cells

**FIGURE 4:**
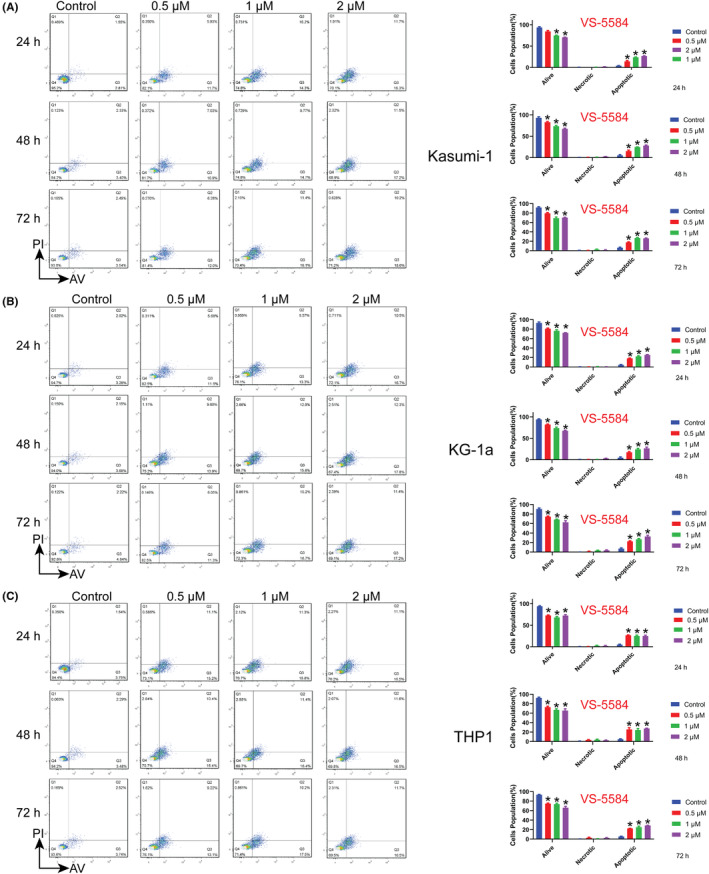
VS‐5584 treatment instigated apoptosis in AML cells. (A–C) varying doses of VS‐5584 treatment were observed to induce apoptosis in (A) Kasumi‐1, (B) KG‐1a, and (C) THP1 cells. The AML cells were subjected to incubation with VS‐5584 for durations of 24, 48, or 72 h. *p<0.05. AML, acute myeloid leukemia cells.

The diminution of cellular proliferative capacity typically encompasses two facets. Firstly, mitotic aberrations result in dysregulation of the cell cycle. Secondly, the decline in viable cells is concomitant with augmented apoptosis. These two factors exert an influence on the destiny of the cell. As previously stated, the administration of VS‐5584 raised the proportions of cells in the G2/M phase. Subsequently, the impact of VS‐5584 on apoptosis in AML cells was evaluated at various intervals. VS‐5584 treatment resulted in a notable rise in the percentage of apoptotic cells (Figure 4A–C). Our results demonstrated that the induction of apoptosis in AML cells by VS‐5584 was contingent upon both the concentration and duration of treatment.

### 3.5 Proteomic analysis of VS‐5584‐treated AML cells

**FIGURE 5:**
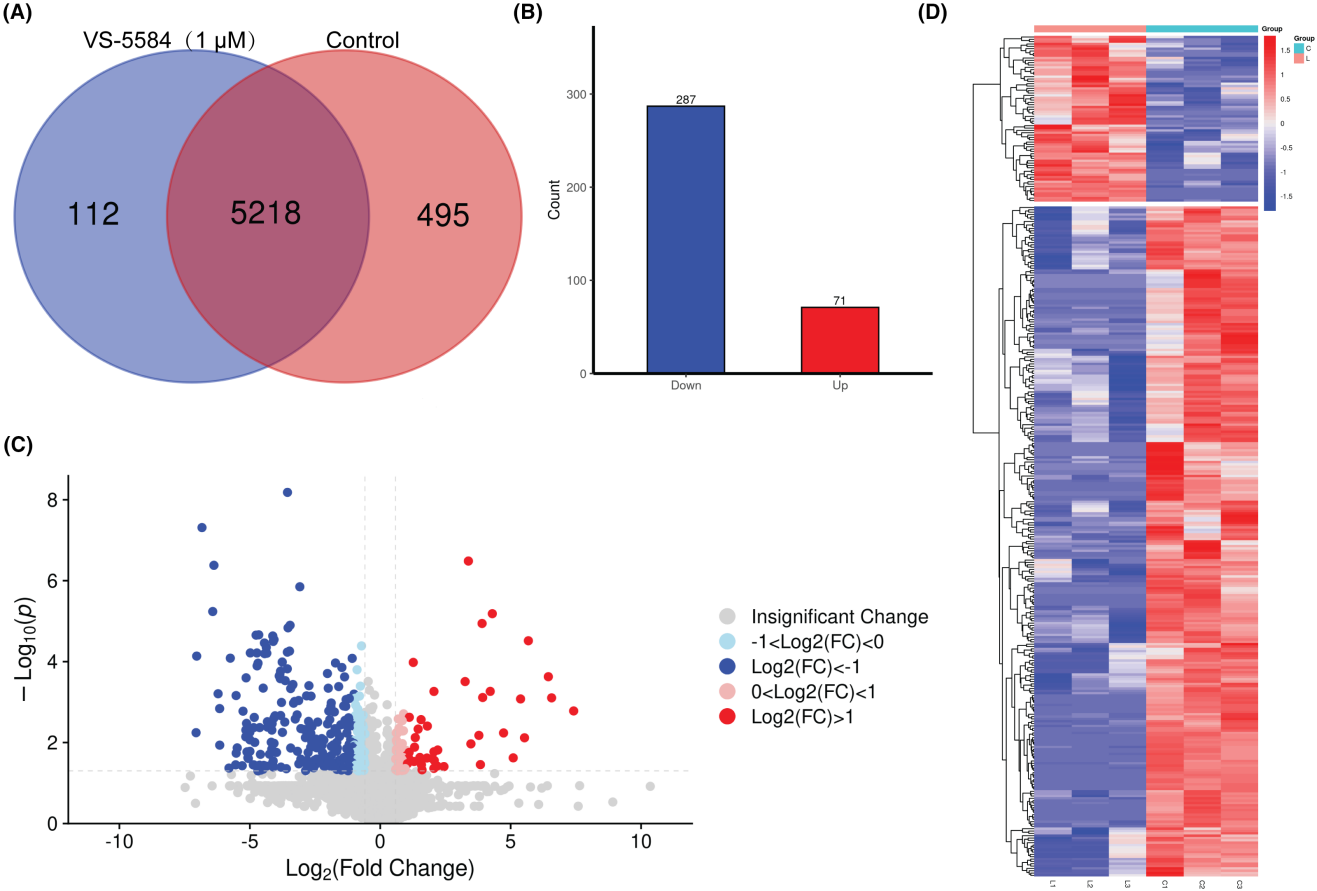
The proteomic analysis of THP1 cells treated with VS‐5584. (A) Venn diagram representing the distribution of differentially expressed proteins in TPH1 cells, wherein TPH1 cells were subjected to 1 μM VS‐5584 or vehicle control for 24 h. (B) Histogram of the number of differential proteins in AML cells with VS‐5584 treatment. (C) Volcano plot displaying differential proteins in AML cells after VS‐5584 treatment. (D) Clustering analysis of differential proteins in AML cells with VS‐5584 treatment.

In order to comprehend the process by which VS‐5584 induced apoptosis and cycle arrest, protein samples were extracted from cells that were subjected to a 24‐h treatment of 1 μM VS‐5584, and subsequently subjected to proteomic analysis (Figure S4). Our proteomic findings revealed that 5218 proteins were identified in both groups. In comparison to the control group, treatment with 1 μM VS‐5584 resulted in the significant down‐regulation of 287 proteins and up‐regulation of 71 proteins (Figure 5A–D). Furthermore, the down‐regulated proteins primarily consisted of cell cycle regulators, such as CCNB1, KI67, and PLK1, while the up‐regulated proteins were mainly composed of DNA demethylation‐related proteins, including USP9X and USP7.

### 3.6 Network map of differential proteins

**FIGURE 6:**
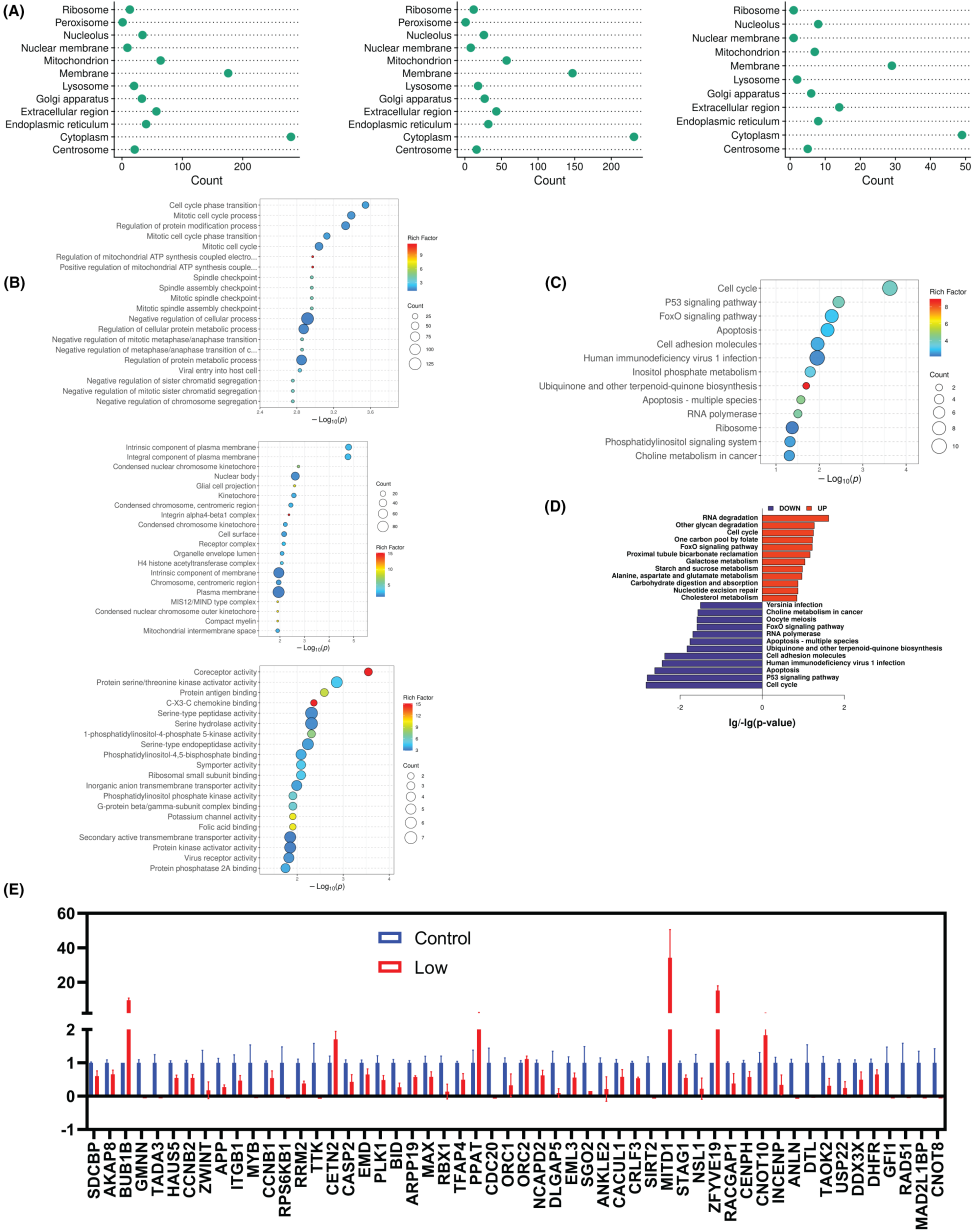
Bioinformatics analysis of differential proteins. (A) The subcellular distribution of differential proteins (left panel), up‐regulated proteins (middle panel), and down‐regulated proteins (right panel). (B) The top 20 enriched Go terms, including biological process (BP; upper panel), cellular component (CC; middle panel), and molecular function (MF; lower panel). (C) Bubble plots showing KEGG enrichment analysis of differential proteins. (D) KEGG pathway enrichment analysis of up‐regulated or down‐regulated proteins. (E) Histogram showing the abundance of proteins associated with cell cycle‐related pathways.

Next, proteomic data underwent bioinformatics analysis, revealing that the differential proteins were primarily localized in the cell membrane and cytoplasm (Figure 6A). The data were further subjected to gene ontology (GO) enrichment analysis, which encompassed three categories: biological process (BP), cellular component (CC), and molecular function (MF). The differential proteins exhibited a notable enrichment in various biological processes, including cell cycle phase transition, mitotic cell cycle process, and mitotic cell cycle. The GO cellular components analysis revealed a significant enrichment of differential proteins in intrinsic component of plasma membrane, integral component of plasma membrane, and condensed nuclear chromosome kinetochore. The molecular function terms were predominantly enriched in coreceptor activity, protein serine/threonine kinase activator activity, and protein antigen binding. (Figure 6B). KEGG pathway analysis revealed that the differential proteins were significantly enriched in cell cycle, p53, FoxO, and apoptosis signaling pathways (Figure 6C). Besides, KEGG analysis of up‐regulated and down‐regulated proteins affected by VS‐5584 was performed respectively. The results demonstrated that the up‐regulated proteins were primarily associated with cell cycle, glycan degradation, and RNA degradation. The down‐regulated proteins are mainly related to cell cycle, P53 signaling pathway, and cell apoptosis (Figure 6D). Additionally, the expression fold difference for proteins related to the cell cycle was examined (Figure 6E). Results of GO and KEGG analysis of up‐regulated (Figure S5) or down‐regulated proteins (Figure S6) are presented in additional files.

### 3.7 SIRT2 inhibitor SirReal2 combined with VS‐5584 inhibited AML cell proliferation

**FIGURE 7:**
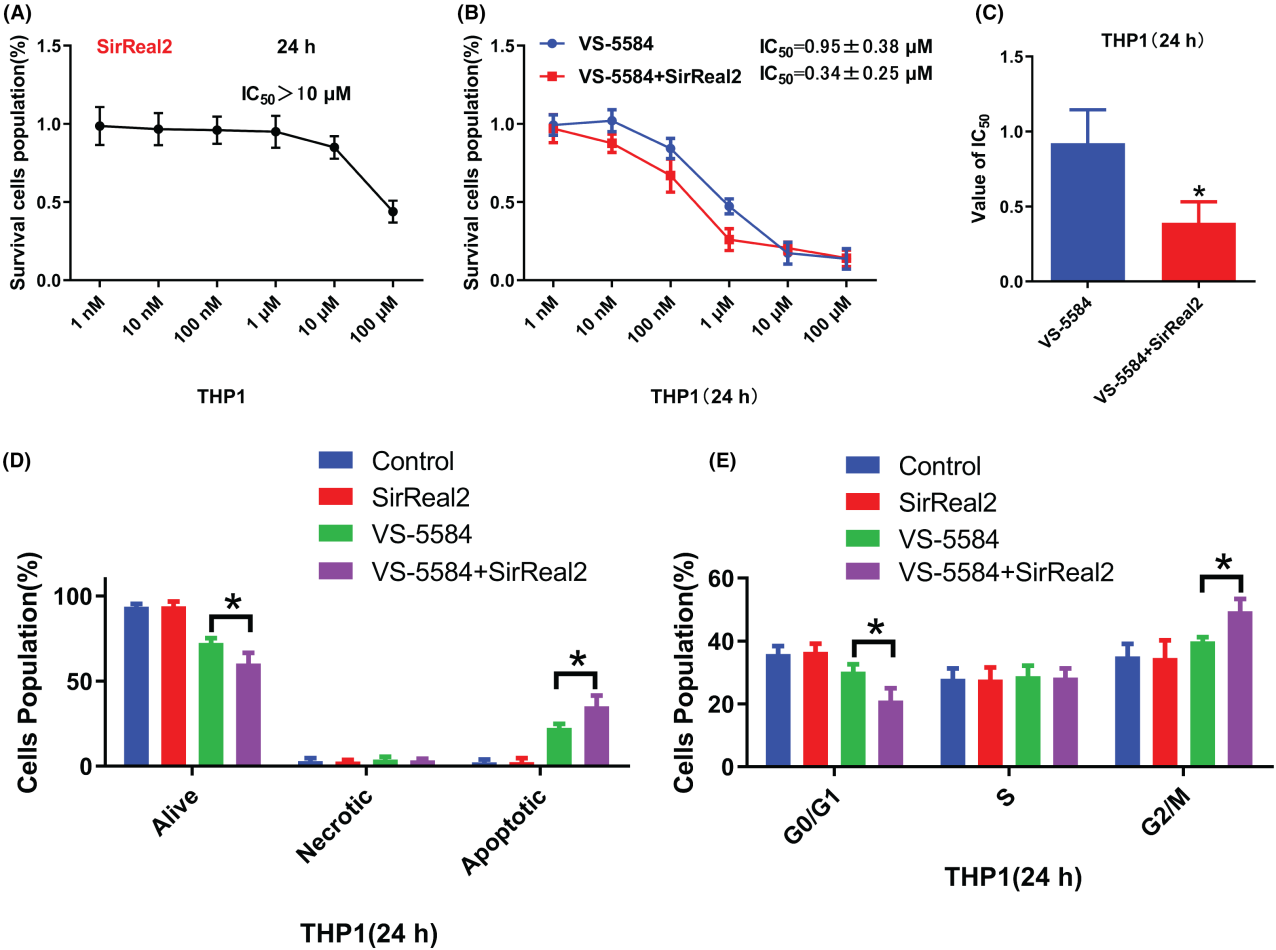
SIRT2 inhibitor SirReal2 combined with VS‐5584 inhibits the proliferation of AML cells. (A) Treatment with SirReal2 alone reduced the proliferative capacity of THP1 cells. (B) The combination of SirReal2 and VS‐5584 inhibited the proliferative ability of THP1 cells. (C) The IC_50_ value of the combined treatment. (D) Effect of drug combination on cell cycle distribution. (E) Effect of drug combination on AML cell apoptosis. *p<0.05, as determined by Student's *t*‐test or one‐way ANOVA with LSD.

Reducing the concentration of small molecule inhibitors is a viable strategy to mitigate their toxicity and side effects, albeit at the cost of potentially compromising their antitumor efficacy. In order to identify promising small‐molecule inhibitors for clinical application, we conducted a literature review^23^, ^24^, ^25^ and identified SIRT2, a member of the NAD+ dependent deacetylases,^26^ as a target for further investigation. Previous work suggested that high SIRT2 expression served as a novel and unfavorable prognostic biomarker in patients with AML.^27^ Here, the experimental protocol involved the administration of 0.5 μM VS‐5584 to AML cells under the aforementioned conditions, followed by the collection of cells for analysis after 24 h. RT‐qPCR and Western blotting analyses verified a marked reduction in both SIRT2 mRNA and protein expression levels in AML cells following VS‐5584 treatment (Figure S7). In addition, AML cells were subjected to treatment with the SIRT2 inhibitor SirReal2, which did not yield significant inhibitory effects at low concentrations (Figure 7A). However, co‐treatment with low concentrations of SirReal2 and VS‐5584 resulted in a reduction of the IC_50_ value of VS‐5584 in THP1 cells (Figure 7B,C). Further experiments involving apoptosis and cell cycle analysis revealed that the combined treatment of 1 μM SirReal2 and 1 μM VS‐5584 was able to significantly increase the proportion of apoptotic cells and induce cell cycle arrest, as compared to cells treated with 1 μM VS‐5584 alone (Figure 7D,E).

### 3.8 VS‐5584 administration restrained the growth of subcutaneous xenografts in vivo

**FIGURE 8:**
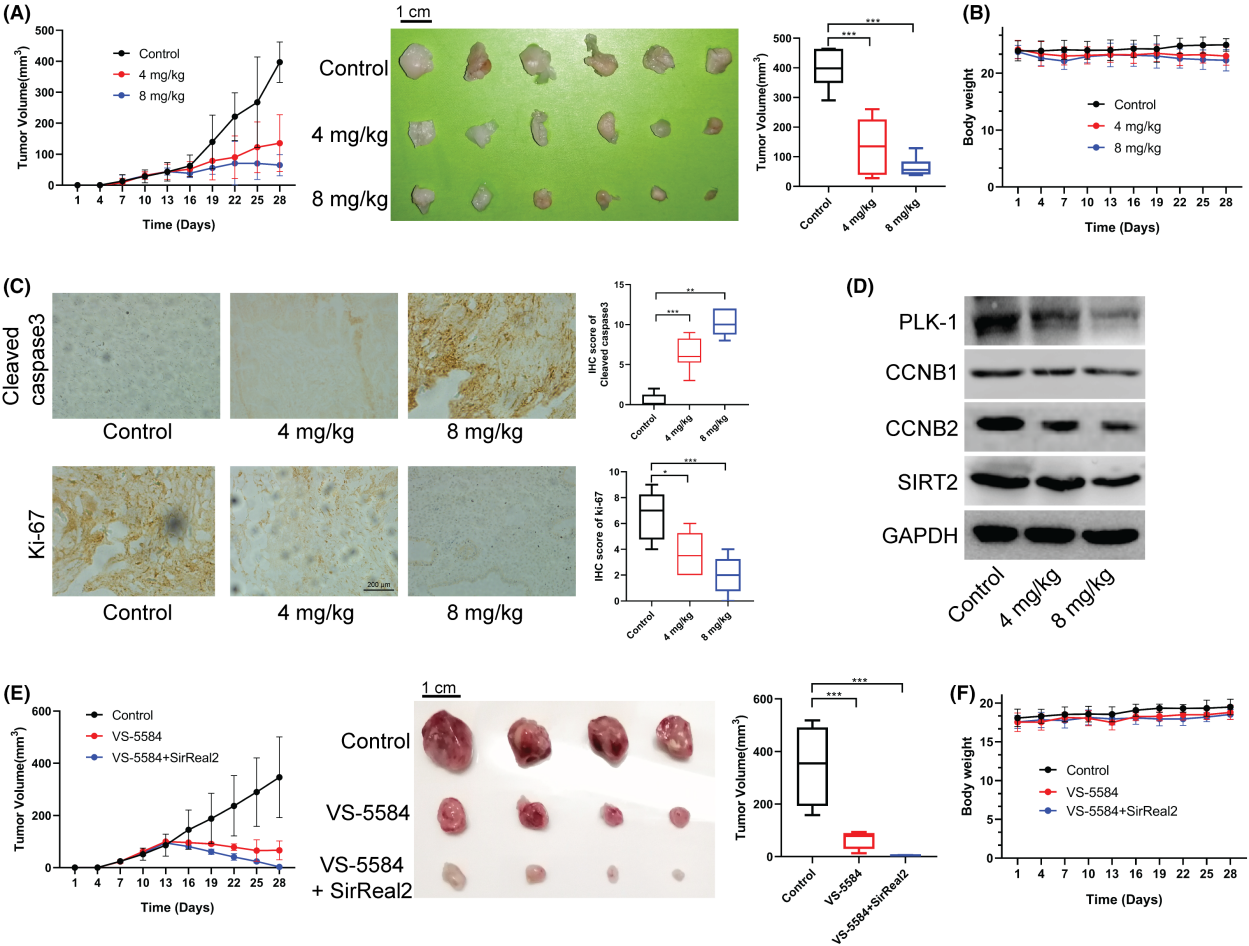
VS‐5584 combined with SirReal2 suppressed the growth of subcutaneous xenografts in vivo. (A) Diameter of subcutaneous tumors; images of subcutaneous tumors; the final volume of subcutaneous tumors. (B) Body weight of mice. (C) Immunohistochemical staining showing the expression levels of cell apoptosis‐ and cell cycle‐related proteins in subcutaneous tumors. (D) Proteins levels of cell cycle‐associated genes in subcutaneous tumors. (E) Diameter of subcutaneous tumors; images of subcutaneous tumors and the final tumor volume. The mice were intraperitoneally administrated with VS‐5584VS‐5584 and SirReal2. (F) Mouse body weight. *p<0.05, **p<0.01, and ***p<0.001, as determined by one‐way ANOVA with LSD post hoc test. AML, acute myeloid leukemia cells.

Animal experimentation plays a crucial role in drug development, as it can prevent erroneous outcomes in in vitro experiments. In order to assess the anti‐AML properties of VS‐5584 in vivo, a mouse xenograft model was created by administering subcutaneous injections of THP1 cells into six‐week‐old nude mice. The results of the study, as evidenced by tumor diameter and volume data, indicated that VS‐5584 effectively suppressed tumor growth in a dose‐dependent manner (Figure 8A). Furthermore, the administration of VS‐5584 did not significantly impact the body weight of the mice (Figure 8B). IHC staining demonstrated that the administration of VS‐5584 significantly increased the expression of Cleaved caspase3, while concurrently reducing the expression of Ki‐67 (Figure 8C). Moreover, the protein levels of PLK‐1, CCNB1, CCNB2, and SIRT2 were observed to decrease in a dose‐dependent manner in tumor tissues following treatment with VS‐5584 in comparison to the control group (Figure 8D). These observations suggest that VS‐5584 treatment instigated apoptosis in AML cells, thereby impeding the proliferation of AML cells. Moreover, Furthermore, the co‐administration of 4 mg/kg of VS‐5584 and 4 mg/kg of SirReal2 resulted in a significant inhibition of subcutaneous xenograft growth compared to the administration of VS‐5584 alone (Figure 8E). Additionally, no significant alterations in the weight of mice were observed during the course of the study (Figure 8F). Additional experiments were conducted to examine the effects of VS‐5584 and SirReal2 on tumor growth using a tail vein injection model. It was found that VS‐5584 combined with SirReal2 led to an extension of the survival time of mice and exhibited significant anti‐tumor effects compared to treatment with VS‐5584 or SirReal2 alone; meanwhile, no differences in body weight were observed among the animal groups (Figure S8).

## 4 DISCUSSION

Despite notable advancements in therapeutic interventions, the five‐year survival rates for patients with AML remain unsatisfactory.^10^ Small molecule inhibitors have garnered attention as a potential treatment option for AML. Nonetheless, it is crucial to prioritize fundamental research endeavors aimed at developing novel therapeutic approaches before clinical implementation.

The PI3K/mTOR pathway is frequently activated in diverse cancer types, as it necessitates prompt response to extracellular signals.^28^, ^29^ The mTOR kinase is responsible for phosphorylating downstream proteins, such as AKT, which are recognized as significant in promoting cancer growth.^29^, ^30^ Small‐molecule inhibitors that have been developed and extensively validated for their tumor‐suppressing effects target the PI3K/mTOR pathway.^13^, ^31^, ^32^ The activation of the PI3K/AKT/mTOR pathway has been reported in AML cells,^13^, ^33^ and targeting this signaling pathway has demonstrated beneficial therapeutic effects on AML both in vitro and in vivo.^13^, ^34^ In this study, bioinformatics analysis consistently demonstrated a significant up‐regulation of PIK3R5 expression in AML tissues. The administration of the PI3K/mTOR inhibitor VS‐5584 resulted in a noteworthy reduction in AML cell proliferation by inducing cell cycle arrest and apoptosis. Additionally, proteomic sequencing confirmed that VS‐5584 treatment modulated the expression of genes related to cell cycle and apoptosis in AML cells. The proteins that exhibited differential expression were predominantly implicated in biological processes encompassing cell cycle progression, mitosis, and apoptosis. Furthermore, these proteins were found to be linked with the P53 signaling pathway, which holds considerable importance in the regulation of cell cycle, apoptosis, and development.^35^, ^36^, ^37^ The activation of the PI3K pathway is known to stimulate cell growth and survival, but its inhibition can result in reduced downstream proliferative signaling, augmented apoptosis signaling, and activation of the P53 signaling pathway.^38^, ^39^ The findings of our investigation furnish substantiation to propose that VS‐5584 manifests a repressive influence on the PI3K/mTOR pathway, concomitantly stimulating the P53 pathway, thereby influencing the proliferation of AML cells.

It is worth noting that small molecule inhibitors are often associated with toxicity and adverse reactions, which can be mitigated by reducing their concentration.^40^, ^41^ Our preliminary findings suggest that VS‐5584 may induce AML cell cycle arrest and apoptosis by modulating signaling pathways involved. In order to delve deeper into the effectiveness of drug combination, we scrutinized genes that were considerably altered following VS‐5584 treatment and recognized SIRT2 as a potential therapeutic target. SIRT2, a NAD(+)‐dependent deacetylase,^26^ is a member of the sirtuin family and has been implicated in various cancers.^42^, ^43^, ^44^ SirReal2, a specific inhibitor of SIRT2, has demonstrated antitumor effects.^43^, ^44^ Prior research has suggested that SIRT2 plays a role in promoting NADPH production through its involvement in the acetylation of G6PD, which in turn regulates AML metabolic reprogramming and facilitates leukemic cell proliferation.^45^ Additionally, another study has shown that inhibiting or deleting SIRT2 can significantly enhance chemosensitivity in MLL‐ENL AML cells.^24^ To date, there exists no substantiated proof that the utilization of SIRT2 inhibitors could enhance the drug sensitivity of AML cells to VS‐5584. However, our research findings have validated that the co‐administration of VS‐5584 and the SIRT2 inhibitor SirReal2 effectively suppresses the proliferation of AML cells in vitro, in contrast to the administration of VS‐5584 alone. Additionally, our study affirms that this drug combination mitigated tumor growth in a pre‐existing xenograft mouse model of AML and extended survival in mice injected with tumor cells via tail vein. The combination of SirReal2 and VS‐5584 did not produce a significant impact on body weight in two mouse models., thereby demonstrating the feasibility and effectiveness of this combination. These findings suggest that the combination of SirReal2 and VS‐5584 may augment antitumor effects and enhance tumor sensitivity to chemotherapy. Nevertheless, additional preclinical investigations are required to ascertain the therapeutic efficacy of this combination in the treatment of AML.

In summary, our investigation has demonstrated that the utilization of PI3K/mTOR inhibitors effectively suppressed the proliferation of AML cells. Additionally, the co‐administration of the SIRT2 inhibitor SirReal2 with the PI3K/mTOR inhibitor VS‐5584 exhibited enhanced anti‐tumor properties against AML cells. These results indicate promising therapeutic approaches and pharmacological agents for the management of AML.

## AUTHOR CONTRIBUTIONS

**Yiming Luo:** Conceptualization (equal); data curation (equal); formal analysis (equal); funding acquisition (equal); investigation (equal); methodology (equal); project administration (equal); resources (equal); software (equal); supervision (equal); validation (equal); visualization (equal); writing – original draft (equal); writing – review and editing (equal). **Haijun Zhao:** Conceptualization (equal); data curation (equal); formal analysis (equal); investigation (equal); methodology (equal); project administration (equal); resources (equal); software (equal); supervision (equal); validation (equal); visualization (equal); writing – original draft (supporting); writing – review and editing (equal). **Jingtao Zhu:** Conceptualization (supporting); data curation (equal); formal analysis (equal); investigation (supporting); methodology (supporting); project administration (supporting); resources (equal); software (equal); supervision (supporting); validation (supporting); visualization (equal); writing – original draft (supporting); writing – review and editing (equal). **Liyi Zhang:** Conceptualization (supporting); data curation (equal); formal analysis (equal); investigation (equal); methodology (equal); project administration (supporting); resources (supporting); software (supporting); validation (supporting); visualization (supporting); writing – original draft (supporting); writing – review and editing (equal). **Jie Zha:** Conceptualization (supporting); data curation (equal); formal analysis (equal); investigation (supporting); methodology (equal); project administration (supporting); resources (equal); software (equal); validation (supporting); visualization (supporting); writing – original draft (supporting); writing – review and editing (equal). **Li Zhang:** Conceptualization (supporting); data curation (equal); formal analysis (equal); investigation (supporting); methodology (supporting); project administration (supporting); resources (equal); software (equal); supervision (supporting); validation (supporting); visualization (supporting); writing – original draft (supporting); writing – review and editing (equal). **Yi Ding:** Conceptualization (supporting); data curation (equal); formal analysis (equal); investigation (supporting); methodology (supporting); project administration (supporting); resources (equal); software (equal); validation (supporting); visualization (supporting); writing – original draft (supporting); writing – review and editing (equal). **Xinyi Jian:** Conceptualization (supporting); data curation (supporting); formal analysis (supporting); investigation (supporting); methodology (supporting); project administration (supporting); resources (supporting); software (supporting); supervision (supporting); validation (supporting); visualization (supporting); writing – original draft (supporting); writing – review and editing (equal). **Junjie Xia:** Conceptualization (equal); data curation (equal); formal analysis (equal); funding acquisition (equal); investigation (equal); methodology (equal); project administration (equal); resources (equal); software (equal); supervision (equal); validation (equal); visualization (equal); writing – original draft (supporting); writing – review and editing (equal). **Bing Xu:** Conceptualization (equal); data curation (equal); formal analysis (equal); funding acquisition (equal); investigation (equal); methodology (equal); project administration (equal); resources (equal); software (equal); supervision (equal); validation (equal); visualization (equal); writing – original draft (supporting); writing – review and editing (equal). **Zhongquan Qi:** Conceptualization (equal); data curation (equal); formal analysis (equal); funding acquisition (equal); investigation (equal); methodology (equal); project administration (equal); resources (equal); software (equal); supervision (equal); validation (equal); visualization (equal); writing – original draft (supporting); writing – review and editing (equal).

## FUNDING INFORMATION

This work was funded by Xiamen Cell Therapy Research Center under grant number 3502220214001.

## CONFLICT OF INTEREST STATEMENT

The authors declare that they have no conflict of interest.

## ETHICS APPROVAL STATEMENT

The animal experiments were approved by the Animal Ethics Committee of The First Affiliated Hospital of Xiamen University.

## Supporting information


Figure S1.

Figure S2.

Figure S3.

Figure S4.

Figure S5.

Figure S6.

Figure S7.

Figure S8.

## Data Availability

All relevant data are within the article. The data that support the findings of this study are available from the corresponding author upon reasonable request.
