# Supplementary figures and images for: SIRT2 inhibitor SirReal2 enhances anti‐tumor effects of PI3K/mTOR inhibitor VS‐5584 on acute myeloid leukemia cells
Source: Cancer Med. 2023 Sep 1;12(18):18901–17. doi: 10.1002/cam4.6480 (PMC10557894; doi:10.1002/cam4.6480)

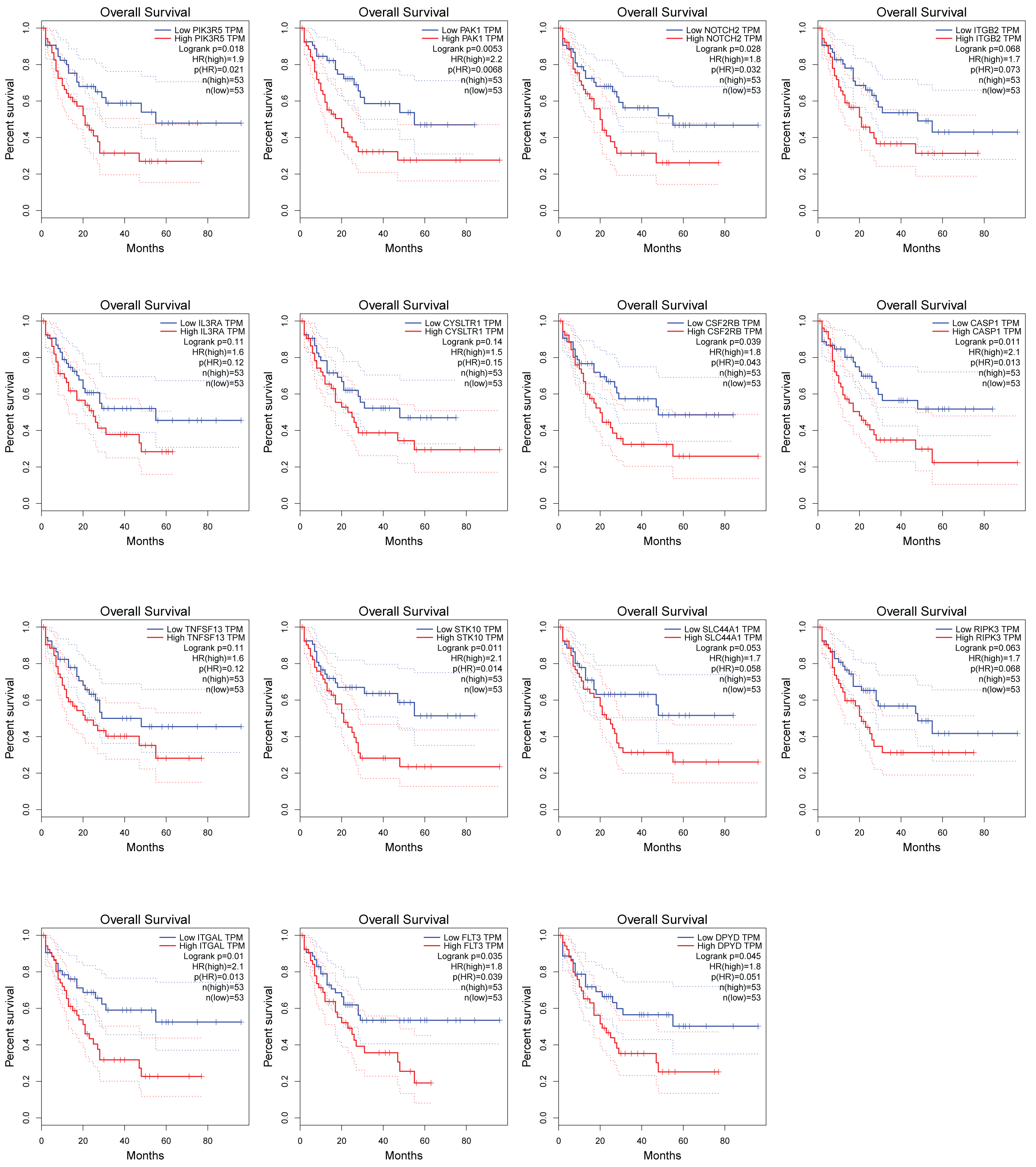

Supplement: Supplementary file 1 — Figure S1. [file CAM4-12-18901-s006.tif]

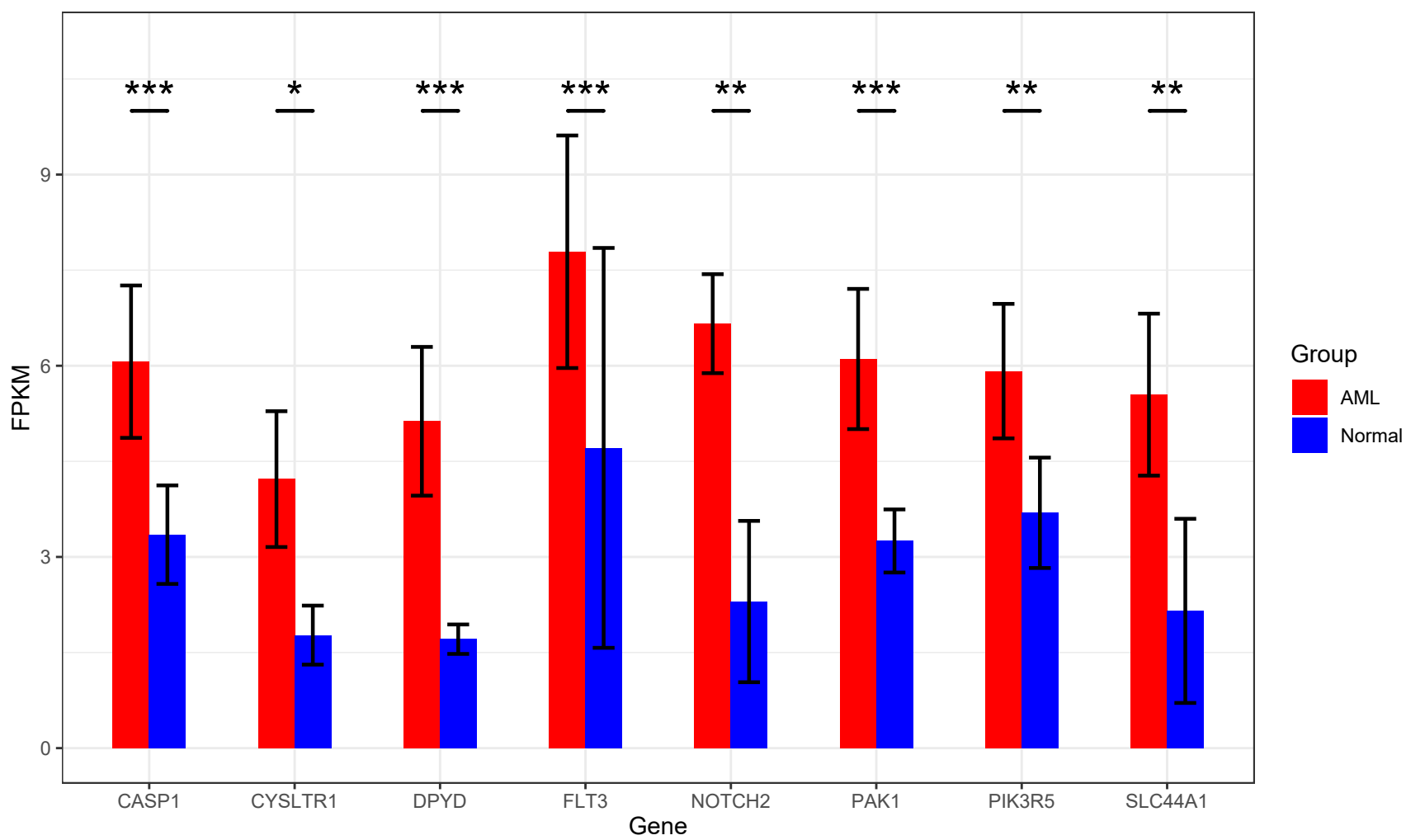

Supplement: Supplementary file 2 — Figure S2. [file CAM4-12-18901-s001.pdf]

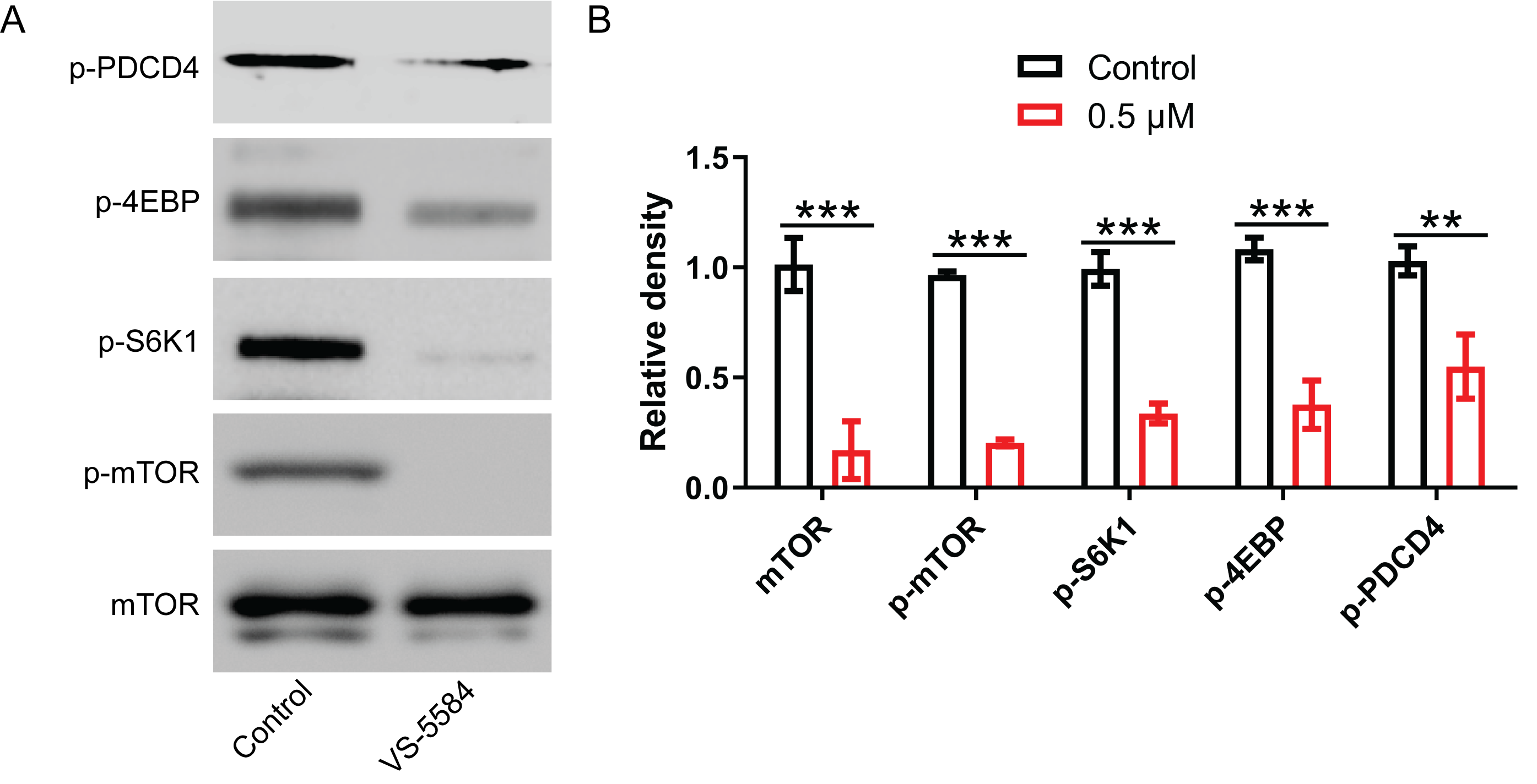

Supplement: Supplementary file 3 — Figure S3. [file CAM4-12-18901-s007.tif]

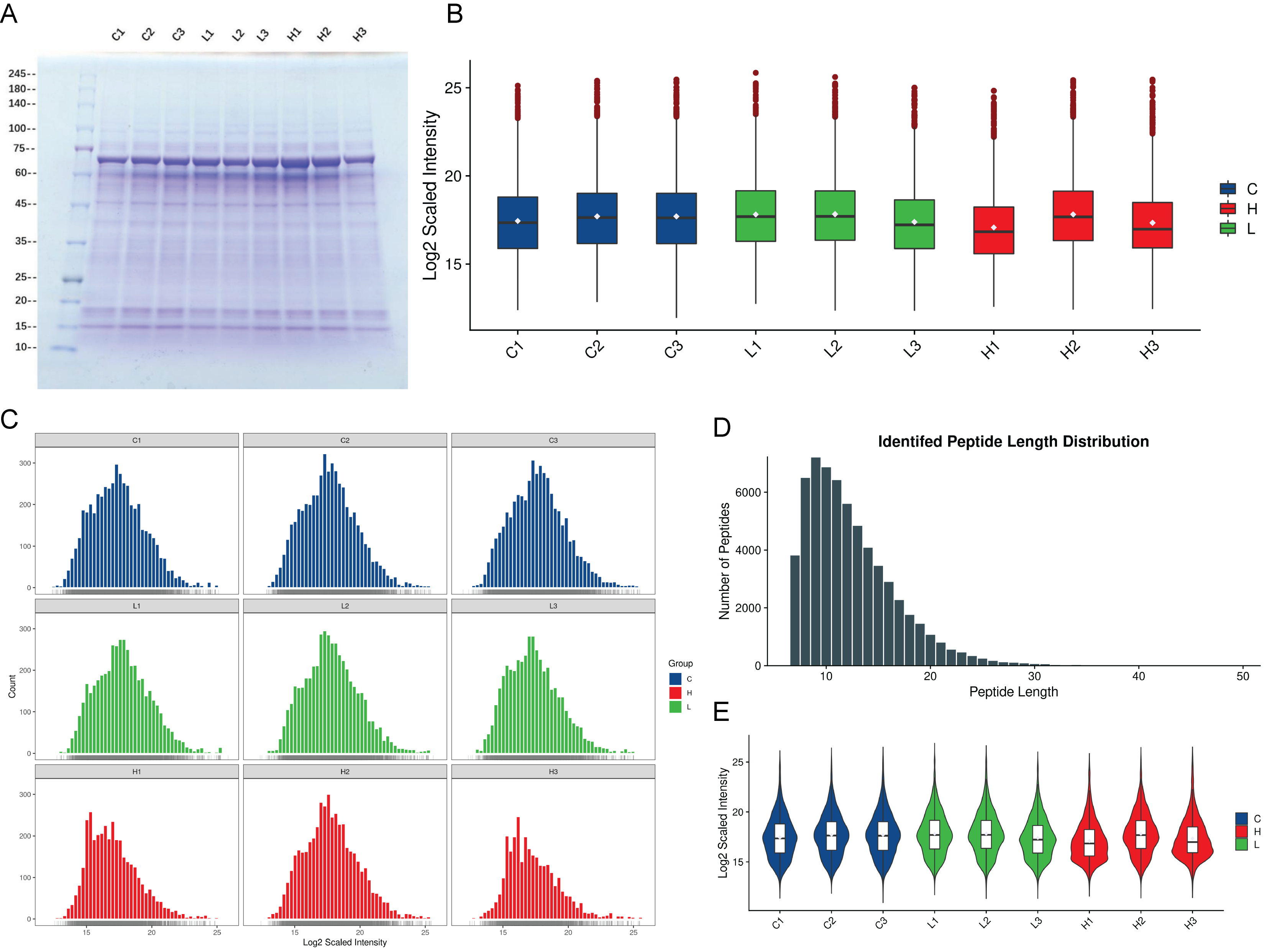

Supplement: Supplementary file 4 — Figure S4. [file CAM4-12-18901-s005.tif]

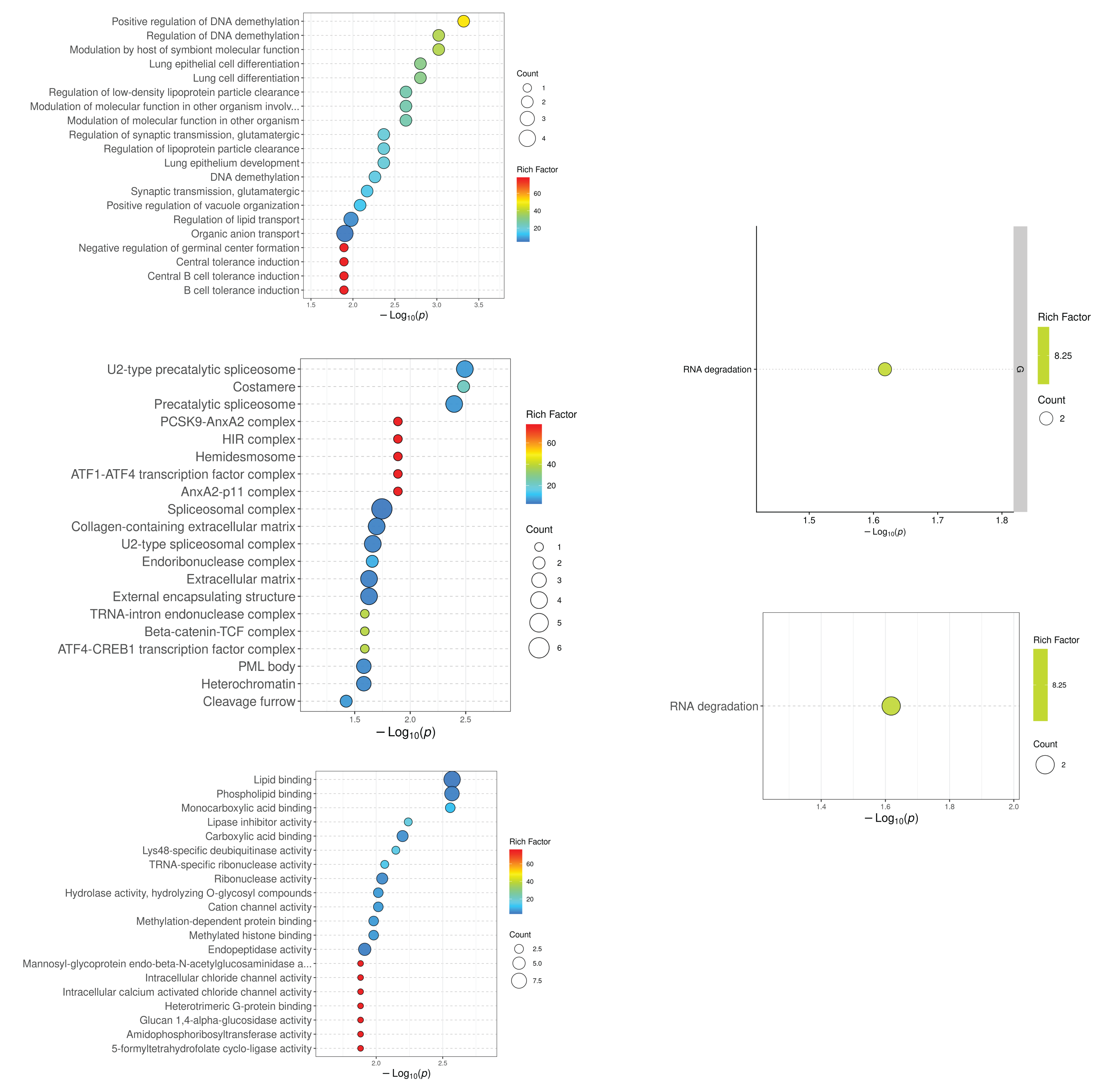

Supplement: Supplementary file 5 — Figure S5. [file CAM4-12-18901-s003.tif]

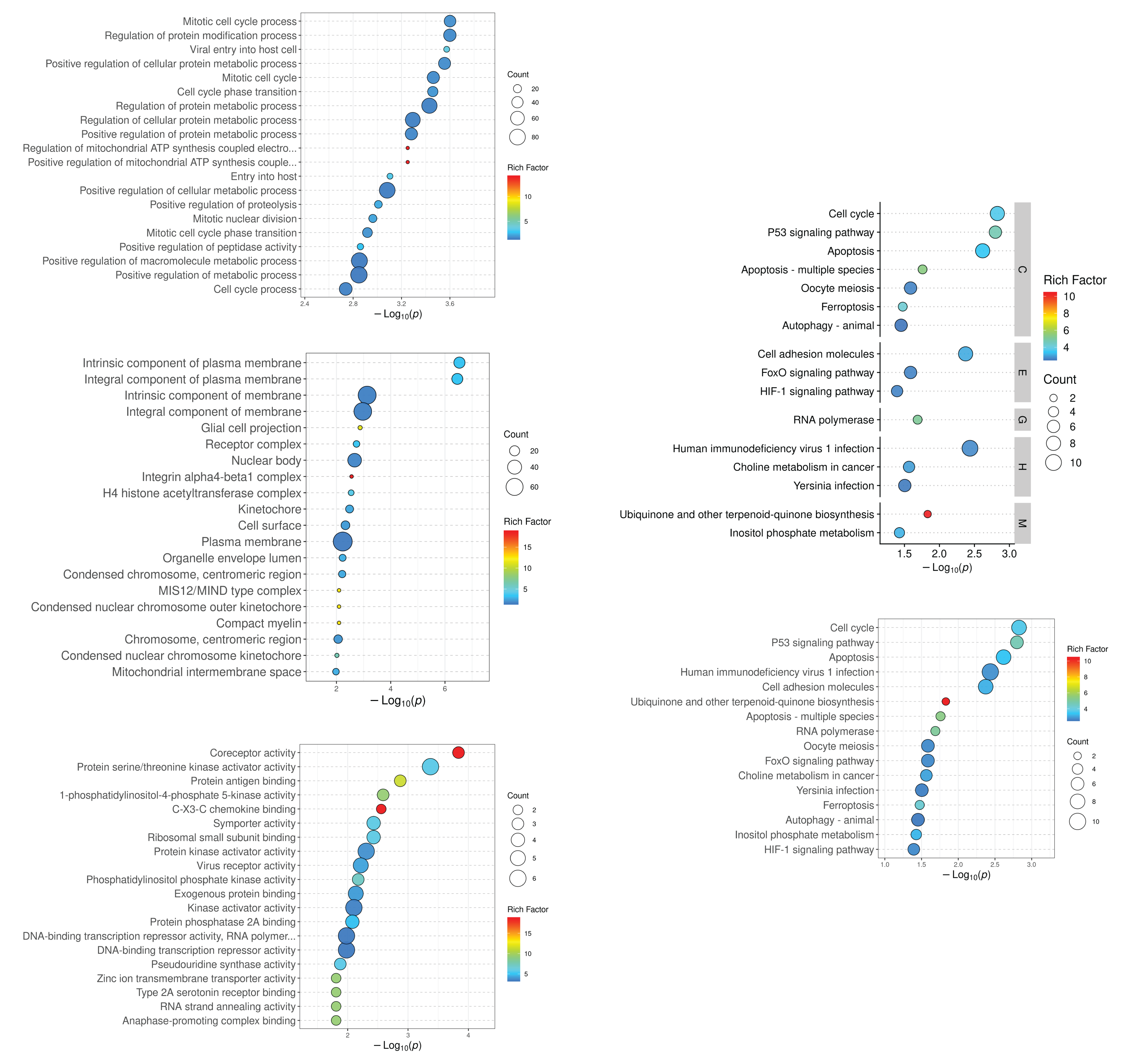

Supplement: Supplementary file 6 — Figure S6. [file CAM4-12-18901-s004.tif]

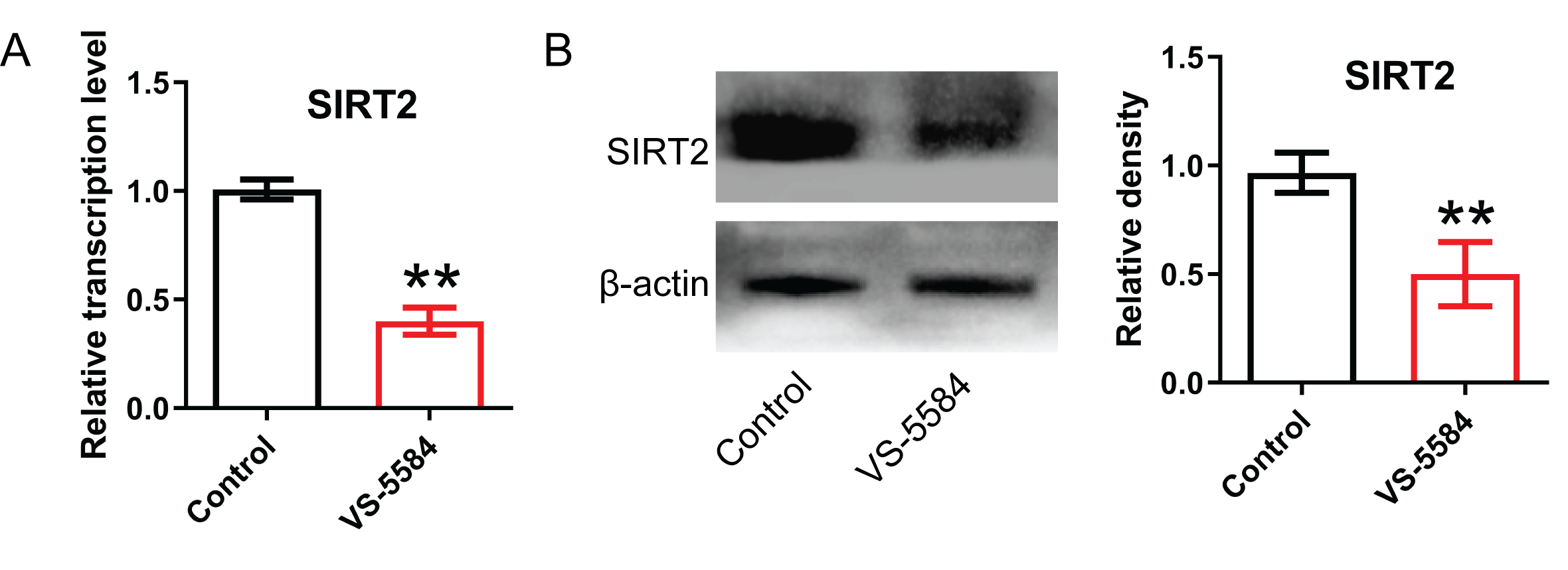

Supplement: Supplementary file 7 — Figure S7. [file CAM4-12-18901-s002.tif]

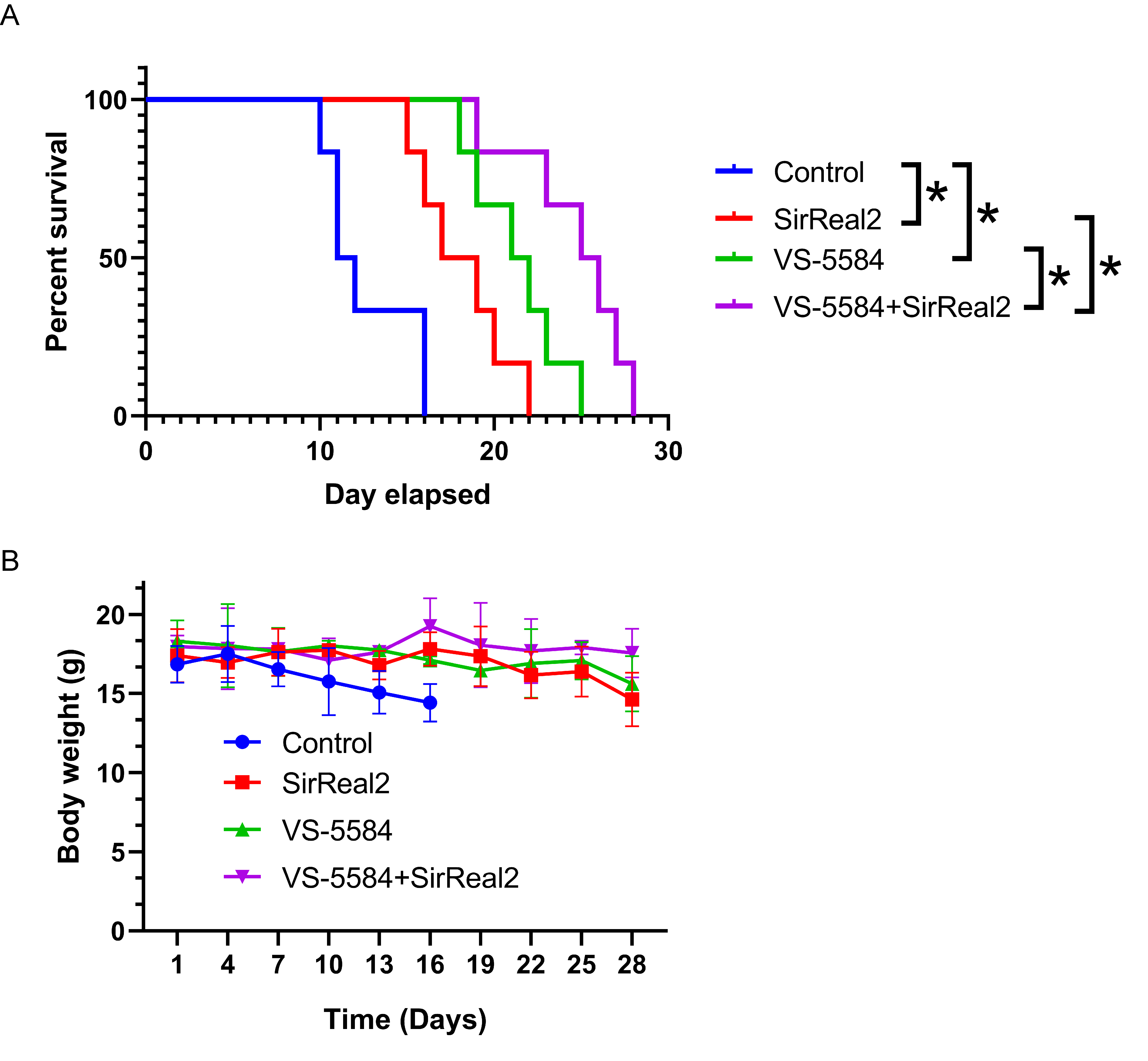

Supplement: Supplementary file 8 — Figure S8. [file CAM4-12-18901-s008.tif]
